# tRFdb: a database for transfer RNA fragments

**DOI:** 10.1093/nar/gku1138

**Published:** 2014-11-11

**Authors:** Pankaj Kumar, Suresh B. Mudunuri, Jordan Anaya, Anindya Dutta

**Affiliations:** 1 Department of Biochemistry and Molecular Genetics, University of Virginia School of Medicine, Charlottesville, VA 22901, USA; 2 Department of Computer Science and Engineering, Grandhi Varalakshmi Venkatarao Institute of Technology (GVIT), Bhimavaram, Andhra Pradesh 534207, India

## Abstract

We have created tRFdb, the first database of transfer RNA fragments (tRFs), available at http://genome.bioch.virginia.edu/trfdb/. With over 100 small RNA libraries analyzed, the database currently contains the sequences and read counts of the three classes of tRFs for eight species: *R. sphaeroides*, *S. pombe*, *D. melanogaster*, *C. elegans*, Xenopus, zebra fish, mouse and human, for a total of 12 877 tRFs. The database can be searched by tRF ID or tRF sequence, and the results can be limited by organism. The search results show the genome coordinates and names of the tRNAs the sequence may derive from, and there are links for the sequence of the tRF and parental tRNA, and links for the read counts in all the corresponding small RNA libraries. As a case study for how this database may be used, we have shown that a certain class of tRFs, tRF-1s, is highly upregulated in B-cell malignancies.

## INTRODUCTION

**Figure 1.**
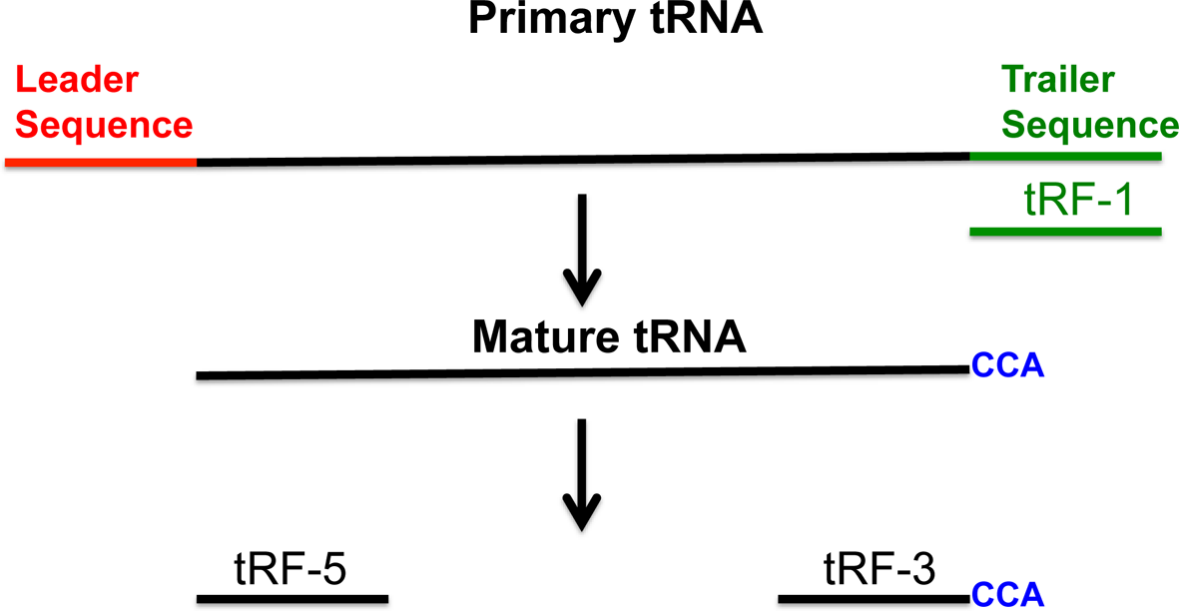
Illustration of primary tRNA, mature tRNA and tRF-5, -3 and -1. tRF-1 (shown in green) is generated from primary tRNA. tRF-5 and -3 are produced from mature tRNA. The tRF-3s always have ‘CCA’ at their 3′ end.

tRNA-derived RNA fragments approaching the size of microRNAs were first appreciated as a class of small non-coding RNA in 2009 by three different laboratories (1–3). Sequences mapping to the 5′ ends of tRNAs (transfer RNA fragment (tRF)-5s), the 3′ ends of tRNAs (tRF-3s) and the trailer sequence (tRF-1s) were observed in LNCap and C4-2 cells, and a tRF-1 was shown to be involved in cell proliferation (Figure 1) (2). tRFs were also found to be present in HeLa nuclei and associated with Argonautes, and present in HEK 293 cells and involved in RNA silencing (1,3). Since then tRFs have been found in all domains of life, and there are now a few reviews cataloging the known functions of tRFs (4,5). Despite this work on tRFs, it is not known how tRF-5s or tRF-3s are generated, and the function of the majority of tRFs is unknown.

tRFs are present in similar abundance to miRNAs, are more evolutionarily conserved and interesting functions for tRFs continue to be discovered, yet the handful of papers on tRFs clearly shows that research on this class of small RNA is lagging behind other small RNAs. This may be due to a general confusion about these sequences and lack of standardized nomenclature, for example the same tRF has been referred to by different names, and a tRF has been mistaken as an miRNA (1,6). In addition, currently there is not a searchable database where researchers can compare tRFs from various experiments.

Because a tRF may be derived from several different tRNAs, and several distinct tRFs may come from the same tRNA, it is not practical to use the name of the parental tRNA in the tRF identifier. As a result, we have decided to improve upon the nomenclature already established in the field (2). In each organism, tRFs are named in the order they are identified, with the first tRF-5 named 5001, the first tRF-3 named 3001 and the first tRF-1 named 1001. In the case of tRF-5s and tRF-3s, there are multiple distinct subclasses (7). When there are two or more tRF-5s that differ only in length: an ‘a’, ‘b’ or ‘c’ is appended for tRF-5s of ∼15, ∼22 or ∼31 bases. All tRF-5as, -bs and -cs share a common seed sequence. Similarly, when there are two distinct tRF-3s mapping to the same tRNA, the tRF-3s of length ∼18 have an ‘a’ appended, while tRF-3s of length ∼22 have a ‘b’ appended. The latter is of particular importance since tRF-3-as and tRF-3-bs have different 5′ ends and therefore different seed sequences.

The field of tRF research is in its infancy and we present the first attempt to classify and tabulate tRF sequences, tRFdb, a database for tRFs. In its current form, the database is a simple way for researchers to view the tRF sequences present in various organisms and compare their read counts in multiple experiments. We hope that our database spurs research into this novel class of small RNA and we invite suggestions from the community on what additions should be added to future versions of tRFdb.

## MATERIALS AND METHODS

### Analysis of the small RNA data

#### Source and processing of small RNA high-throughput sequencing data

The small RNA high-throughput sequencing data were downloaded from the GEO (http://www.ncbi.nlm.nih.gov/geo/) and NCBI SRA databases (http://www.ncbi.nlm.nih.gov/sra). Information about the tRNA genes for species *Rhodobacter sphaeroides* (Bacteria; ATCC_17025), *Schizosaccharomyces pombe* (schiPomb1), Drosophila (dm3), *Caenorhabditis elegans* (ce6), Xenopus (xenTro3), zebra fish (Zv9), mouse (mm9) and human (hg19) was either downloaded from the ‘Genomic tRNA database’ (http://gtrnadb.ucsc.edu) or NCBI (http://www.ncbi.nlm.nih.gov/). We extracted mature tRNA genes from the same genome assembly on which the tRNA gene coordinates were built and added ‘CCA’ at the end. In addition to that we also add 50 bases downstream sequences to find the tRFs generated from the tRNA trailer sequences. The genomic sequences were extracted based on the strand information of tRNA gene transcription. A species-specific tRNAdb blast database was built to query the small RNA sequences and the small RNAs were mapped using BLASTn (8). We considered only those alignments where the query sequence (small RNA) was mapped to the database sequence (tRNA) along 100% of its length with 100% identity. To eliminate any false positives, the small RNAs that mapped on to the ‘tRNAdb’ were again searched against the whole genome database using blast search excluding the tRNA loci. Only those small RNAs that mapped exclusively to tRNA loci were included as probable tRFs.

#### Quality filter to remove random degradation products of tRNA genes

**Figure 2.**
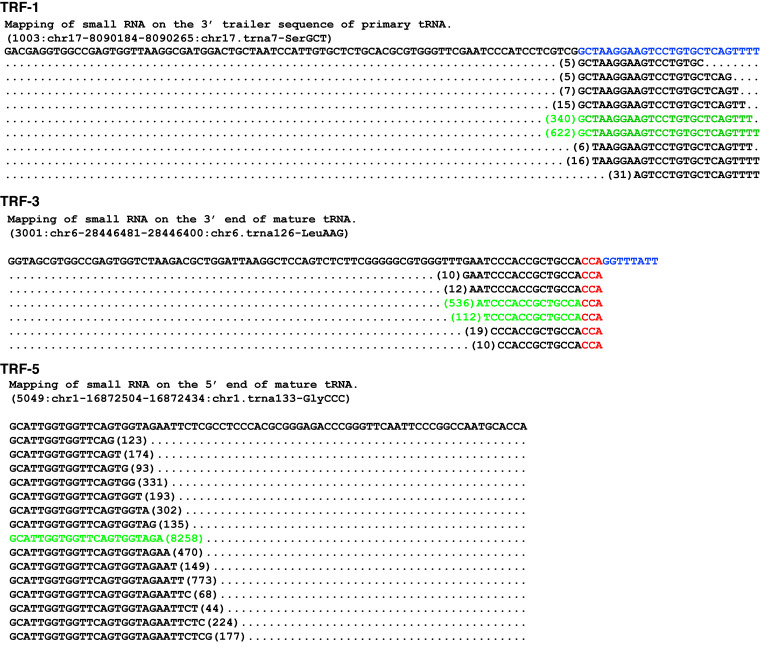
The patterns of small RNA deep sequencing reads (GSM416733) mapping to tRNA genes. Examples of small RNA reads that mapped to specific tRNAs are shown. More than 80% of reads for a given tRF-1, -3 or -5 represent one or two most abundant reads and this is the read that is included as the main tRF sequence in the database. The most abundant clones are shown in green. Upper: tRF-1-like sequences mapping to the primary tRNA chr17.trna7-SerGCT. Middle: tRF-3-like sequences mapping to the mature tRNA chr6.trna126-LeuAAG. Lower: tRF-5-like sequences mapping to the mature tRNA chr6.trna126-LeuAAG.

The ends of the small RNA mapped on tRNA genes was used to assess the significant enrichment of any mapped small RNA on tRNA. The small RNA mostly (>90% of total mapped reads on individual tRNA) mapped on three specific regions: extreme 5′ end (tRF-5), extreme 3′ end (tRF-3) of mature tRNA and 3′ trailer region (tRF-1) of primary tRNA genes. Therefore, tRFs mapped only to these specific locations were considered for building of database. As shown in Figure 2, for each tRF, there is one or two most abundant RNA sequenced (e.g. the tRF-5 ‘GCATTGGTGGTTCAGTGGTAGA’ was sequenced at 8258 reads per million (RPM)) accounting for more than 80% of the reads mapping to that site. This distinguishes the main tRF from other low abundance products created by nucleases digesting the main tRF, or possibly from random degradation of tRNAs and tRFs.

## RESULTS

### tRFs are non-random tRNA fragments

The presence of one highly abundant clone for each tRF (tRF-5, -3 or 1-series) (Figure 2) gives support that the tRF is generated from an individual tRNA with specificity. Furthermore the 5′ end of tRF-1 exactly corresponded to the base immediately downstream from the RNaseZ cleavage site and the 3′ end of tRF-1 contained Pol III transcription termination motifs, also supporting the view that tRF-1s are specific and stable small RNA generated from processing of the precursor tRNA. Interestingly, the leader sequence of the precursor tRNA was rarely found with the abundance of the other tRFs.

For humans and mice, tRF-5s are mainly 15, 22 and 32 nts, whereas tRF-3s are 18 and 22 bases long, while in other organisms tRF-5s and tRF-3s do not contain clear subclasses (7). Depending upon the distance of the transcription termination site, a variable length of small RNA with 3′ poly ‘U’ tract is generated for different tRNA genes (2,9) and therefore the tRF-1s are more variable in length. All the tRFs that originate from the 5′ end of mature tRNA were found to start with the first base of the mature tRNA, indicating that these tRFs are generated after the removal of the leader sequence from the pre-tRNA. The tRFs, which originate from the 3′ end of mature tRNAs, always had a ‘CCA’ at their 3′ end (2,9). All the tRF-1s exactly start (5′ end of tRF) with the first unpaired base (discriminator base) at the 3′ end of the acceptor stem of pre-tRNA while the end base (3′ end of tRF) is within a RNA pol III transcription termination sites (poly ‘U’ tract), indicating that tRF-1s are generated by tRNA 3′ processing enzymes during tRNA maturation (2).

### Exploration of database

A snapshot of the database search page is shown in Supplementary Figure S1. The output can either be viewed online or the user may download the output after searching on the selected parameters. The database can be searched either by tRF type (tRF-5, -3 or -1) or tRF-ID (5001, 5001a, 5001b, 5001c, 3001, 3001a, 3001b, 1001 etc.). The database has an option to select the species of interest or search all species together. The output is displayed as a table: tRF-ID, organism name, tRF type, tRNA gene co-ordinate, tRNA gene name and hyperlinks to the tRF sequence itself and the small RNA experiments that detected the tRF. The ‘Sequence’ hyperlink provides the length and sequence of a given tRF and the originating tRNA gene. The ‘Experiment’ hyperlink provides the GEO ID of the experiments where the tRF was identified, the abundance of the tRF in those datasets and their source (cell line name or tissue). Sublinks from the ‘Experiment’ page provide additional information: the ‘View Alignment’ hyperlink from each experiment displays the alignments (and read frequencies) of all short RNAs that map to the tRNA gene that yields the tRF (similar to Figure 2). The ‘View Graph’ link displays a summary of the alignments as a histogram of the number of times a base on the tRNA gene is represented in the short RNA library. An example of such a histogram is in Supplementary Figure S2 and demonstrates graphically that the tRFs included in tRF-db are extensively enriched relative to other short fragments derived from that tRNA gene. When a tRF-5 or tRF-3 maps to multiple tRNA genes, all the tRNA genes with identical sequence in the relevant area are assigned as a potential source of the tRF. tRF-1s, in contrast, are generated from the 3′ trailer sequence of the primary tRNA and are mostly unique to individual tRNA genes.

Finally, the Help tab provides the help-page that explains the basic concept about tRFs and how to explore and understand the output of the tRFdb. The Statistics tab provides the number of unique tRFs and number of library analyzed for each of the species. Feedback button allows users to provide feedback to the database administrator for any missing or dead link, trouble-shooting or to provide new information or suggestion for the improvement of database.

### Future improvements in tRF-db

The ‘Sequence search’ function already allows the user to identify all tRFs from all species with the exact sequence, even if the tRF-IDs are different between species. In the short term, we will standardize the tRF-IDs such that a given ID will identify tRFs with the same sequence in different species, much like mmu-miR-206 and hsa-miR-206, which identify microRNAs with the same sequence in mouse and human, respectively. Another improvement will be to allow mismatches in the ‘Sequence search’ function to identify closely related tRFs. This will allow us to group tRF-5s and -3s with the same seed sequence into the same tRF families. In the slightly longer term, this database will be expanded to include targets of tRFs predicted by experimental data such as PAR-Clip or CLASH. We also plan to include the stress-induced tRNA halves (tiRs) that have been widely reported in the literature (10). Finally we propose to provide links to current and future publications that contain functional information on the tRFs as they appear.

### tRFs are differentially expressed: a case study of tRFs in normal and cancer B cells

We discovered a very intriguing difference in the expression of tRFs in small RNA isolated from normal and malignant human B cells (11). The small RNA was isolated from each of the four subsets of B cells (naive, germinal center, memory and plasma cell) from normal human subjects in two replicates (from two different individuals). In the same study, small RNAs were isolated from human B-cell-derived tumors for each B-cell subset. tRF-1s, as a class, were more abundant in the malignant compared to normal in all the subsets of B cells. In contrast, the abundance of tRF-5s or tRF-3s was not significantly different in normal and malignant B cells. The differentially expressed tRFs between the normal and malignant B cells that were detected at >20 RPM are shown in Supplementary Figure S3A and B (included in the database). Many of the individual tRF-1s were 100–1000-fold more abundant in the malignant B cells compared to the normal B cells. However, specific tRF-5s or tRF-3s did not exhibit a similar induction in cancer B cells as shown in Supplementary Figure S4 (included in the database). In fact, several tRF-5s or -3s were equal or less abundant in the malignant B cells. Thus, the induction of tRF-1s in malignant B cells is not simply a reflection of higher metabolism of tRNAs in the cancer cells. These differentially expressed tRF-1s need to be further tested to discover the significance of their high expression in tumor cells and to determine if they can be used as biomarkers for B-cell malignancy.

## DISCUSSION

tRFs are a newly discovered class of micro-RNA-sized small RNAs that are highly abundant in different human and mouse cell lines, mouse tissues and organisms ranging from bacteria to humans. Individual tRFs are generated with precise ends and are not degradation products of the tRNAs. Mutation of different components of the miRNA biogenesis pathway does not affect tRF levels (7). In human HEK293 cells, tRF-5s and tRF-3s are associated with Argonautes 1, 3, and 4 as evidenced by PAR-CLIP data (7) (included in the database).

Seven to eight base-long seed sequences at the 5′ ends of miRNAs have to be complementary to the target gene 3′UTR to suppress the expression of the target gene. Considering the miRNA-like binding of tRFs to Ago proteins, and CLASH data indicating the tRFs bind to target mRNAs that are complementary to the 5′ seed sequences of tRFs (7), it is important to note that though tRF-3 and tRF-1 have similar 3′ ends (CCA in case of tRF-3 and poly ‘U’ in case of tRF-1) (2), their 5′ ends have much more diversity, thus targeting different 3′UTR regions. However, much like miRNAs, we expect to subclassify the tRFs into tRF families based on similarities of the 5′ seed sequences, and this will be added to the database later.

It has been suggested recently that only highly expressing miRNAs are functional in mammals (12). Thus, it is noteworthy that many of the tRFs are sequenced at an abundance comparable to that of many abundant microRNAs. For example, in the GSM416733 dataset, the five most abundant microRNAs and their RPM are: hsa-miR-106b-5p (4101), hsa-miR-103a-3p (5222), hsa-miR-20a-5p (5316), hsa-miR-16-5p (9630) and hsa-miR-17-5p (9883). In comparison, the RPM of the two most abundant tRF-5s, -3s and -1s in the same dataset are: tRF- 5014a (8226), tRF- 5030b (7890), tRF- 3008a (8132) and tRF- 3008b (7313), tRF-1032 (2341) and tRF-1037 (3117)!

tRF-3s associate with PIWI protein Twi12, an Argonaute family protein in Tetrahymena that does not have slicer activity like Ago-2 (13). Many of the human tRF-3 and -5s also bind strongly with Ago1-3-4 compared to Ago-2 protein (7). Since Ago-1-3-4 also do not have slicer activity, the tRFs may be involved in as yet undiscovered functions unique to the non-slicer Ago proteins. It is also possible that the high association of tRFs with non-slicer Argonaute family of proteins may be to sequester the tRFs to prevent them from interfering with Ago-2 containing RNA-induced silencing complexes. This database will help researchers explore how tRFs contribute to gene-regulation circuits through their association with Argonaute proteins and inspire a search for other proteins that associate with and are effectors of this enigmatic class of non-micro-short RNAs.

## SUPPLEMENTARY DATA

Supplementary Data are available at NAR Online.
